# Homophobic Abuse & LGBTQ+ Well-being in the Acute Psychiatric Setting

**DOI:** 10.1192/bjo.2022.397

**Published:** 2022-06-20

**Authors:** Edward Kane, Miranda Lloyd, Maeve Malley, Thomas Fox

**Affiliations:** Oxleas NHS Foundation Trust, London, United Kingdom

## Abstract

**Aims:**

Homosexuality was declassified as a mental illness in 1973 however LGBTQ+ (lesbian, gay, bisexual, transgender, queer inclusive) service users still face discrimination within modern mental health services. This project assessed homophobia and LGBTQ+ abuse among service users on an acute male psychiatric ward. Our aims were to quantify the incidence of abuse, to explore staff attitudes toward LGBTQ+ abuse and to identify targets to improve LGBTQ+ service users’ experience. We hypothesised that incidents of abuse are common and not always challenged or escalated using appropriate channels.

**Methods:**

Using a mixed methods approach we explored staff perceptions of LGBT+ abuse: quantitative data were generated from a questionnaire survey and qualitative data from a focus group.

Rates of homophobic incidents were assessed by analysing clinical documentation from two inpatient samples (n = 20), covering 2020–21 and 2021–22.

**Results:**

Analysis of clinical documentation found three incidents from the 2020–21 sample and two from 2021–22; only one of these was reported via DATIX.

The survey captured the views of the ward team including nurses, healthcare assistants (HCAs), doctors and psychologists (response n = 13). Staff attitudes towards LGBTQ+ were rated as “positive” by 77% of responders and “neutral” by 23%; 100% stated it was their professional duty to respect and protect LGBTQ+ clients. Almost two-thirds (62%) had witnessed homophobia on the ward however a similar proportion (61%) had never directly challenged homophobia. Whilst all staff felt able to care for LGBTQ+ clients, and all were familiar with key LGBTQ+ terminology, only 50% felt they had received adequate training to fully support LGBTQ+ clients.

The focus group identified a nursing “lead” for LGBTQ+ issues and agreed to incorporate a “diversity statement” into ward admission rules. LGBTQ+ visibility measures were promoted including LGBTQ+ posters across the ward and staff uptake of the Rainbow Badge Initiative.

**Conclusion:**

Our findings suggest homophobia is prevalent in the male inpatient psychiatric setting and management is suboptimal. Enhanced LGBTQ+ training is required to support staff to challenge every homophobic incident and escalate appropriately.

Simple steps to increase LGBTQ+ visibility are feasible and popular among staff. Future work should assess the impact of such interventions, however measuring change may be hampered by underreporting.

Further evaluations are needed to assess female wards and patient perspectives to build a full picture of inpatient LGBTQ+ abuse.

